# Pollution Characteristics and Spatial Distribution of Heavy Metals in Coal-Bearing Sandstone Soil: A Case Study of Coal Mine Area in Southwest China

**DOI:** 10.3390/ijerph19116493

**Published:** 2022-05-26

**Authors:** Dongping Deng, Yong Wu, Yi Sun, Bangzheng Ren, Lei Song

**Affiliations:** 1 College of Environment and Civil Engineering, Chengdu University of Technology, Chengdu 610059, China; ddp900304@163.com (D.D.); rbz3796@163.com (B.R.); songlei21@mails.ucas.ac.cn (L.S.); 2 State Key Laboratory of Geohazard Prevention and Geoenvironment Protection, Chengdu 610059, China; 3 School of Civil Engineering, Southwest Jiaotong University, Chengdu 610031, China; sunnys90@163.com

**Keywords:** coal-bearing sandstone, heavy metal pollution, soil, multivariate statistics, spatial distribution

## Abstract

Soil pollution in coal mining areas is a serious environmental problem in China and elsewhere. In this study, surface and vertical profile soil samples were collected from a coal mine area in Dazhu, Southwestern China. Microscopic observation, concentrations, chemical speciation, statistical analysis, spatial distribution, and risk assessment were used to assess heavy metal pollution. The results show that the weathering of coal-bearing sandstone and mining activities substantially contributed to soil pollution. The concentrations of Fe, Ni, Cu, Zn, Mn, Cd, Hg, and Pb exceeded their background values. Cd caused the most intense pollution and was associated with heavily–extremely contaminated soils. The residual fraction was dominant for most metals, except Cd and Mn, for which the reducible fraction was dominant (Cd: 55.17%; Mn: 81.16%). Zn, Ni, Cd, and Cu presented similar distribution patterns, and Hg and As also shared similar distribution characteristics. Factor 1 represented anthropogenic and lithologic sources, which were affected by mining activities; Factor 2 represented anthropogenic sources, e.g., fertilizers and traffic pollution; and Factor 3 represented the contribution of metals from soil-forming parent material. More than half of the study area had high pollution risk and was not suitable for vegetable cultivation.

## 1. Introduction

Coal accounts for approximately 76% of China’s primary energy consumption, and is predicted to remain the country’s primary energy source for several years [1]. The coal mining industry is an important source of heavy metals in the environment, and is a major contributor to soil pollution [2,3]. The accumulation of tailings and transportation of coal, including the establishment of a large number of chemical plants, can lead to heavy metal enrichment in the soil and affect the local ecological environment [4,5]. This threatens the land productivity, ecological integrity, and ecological security of nearby areas [6,7]. Through erosion, weathering, and leaching of tailings, the metals present in the tailings can enter the surrounding groundwater, streams, sediments, and soil [8]. Moreover, in addition to causing environmental degradation, heavy metal pollution also threatens soil ecosystems and human health through food chain contamination [9,10]. High concentrations of heavy metals reduce the diversity of soil bio-communities, lead to plant toxicity, and affect agricultural productivity [11,12]. Heavy metals exist in the soil in different fractions (exchangeable, reducible, oxidizable, and residual fractions). The exchange fraction in heavy metals is easier for the plants to absorb, making it more toxic [13]). Therefore, heavy metals can threaten food security and human health through the water supply and food web [14,15].

In Southwest China, especially in the Sichuan Basin, most coal is mined in Triassic sandstone strata. The stratum is generally exposed on the surface or buried at a shallow depth and forms the unique Quaternary eluvium soil in the mining area after weathering. Weathering of parent rocks produces the soil primary and secondary minerals, such as quartz, calcite, and montmorillonite. The minerals are different in particle size, cation exchange capacity, metal species, etc., which endows soils with corresponding properties [16]. Under the influence of coal accumulation, coal and its strata in the sedimentary environment are enriched in heavy metals. With the weathering of coal-bearing sandstone, heavy metals migrate into the soil and enrich it during soil formation [17]. This soil formed by weathering of coal-bearing sandstone is widely distributed, which makes the analysis of this soil more representative. Moreover, coal mining drives the development of the local industry and agriculture, which also leads to the enrichment of heavy metals in the soil [18]. Therefore, the accumulation of heavy metals in the soil, especially in the weathered area of coal-bearing sandstone, should be investigated.

Researchers have adopted various factors and methods, including geoaccumulation index, pollution factor, and enrichment factor, to determine the degree of soil pollution [19]. The qualitative and quantitative chemical speciation of heavy metals in the soil is an important basis to clarify its migration and transformation and evaluate its potential environmental impacts [20]. Geographic information system (GIS) technology is widely used to quantify the spatial distribution of metals and identify pollution sources with low costs [21]. The availability and risks associated with soil pollution by heavy metals should be investigated for the development of reliable pollution management strategies.

To the best of our knowledge, this is the first study to investigate the characteristics of this typical soil through geological, environmental, and statistical analyses. Therefore, based on the heavy metal contents in the surface and vertical profiles of soil samples from a coal mine area in Dazhu, the objectives of this study were (1) to investigate the influence of weathering of coal-bearing sandstone on heavy metals in the soil; (2) characterize the pollution of heavy metals in the soil; and (3) analyze the correlation and spatial distribution of heavy metals in the soils of the study area. The findings can help clarify the environmental impacts of coal mining and closed mines as a basis for the development of mitigation and prevention measures by local stakeholders and authorities.

## 2. Materials and Methods

### 2.1. Site Description

**Figure 1:**
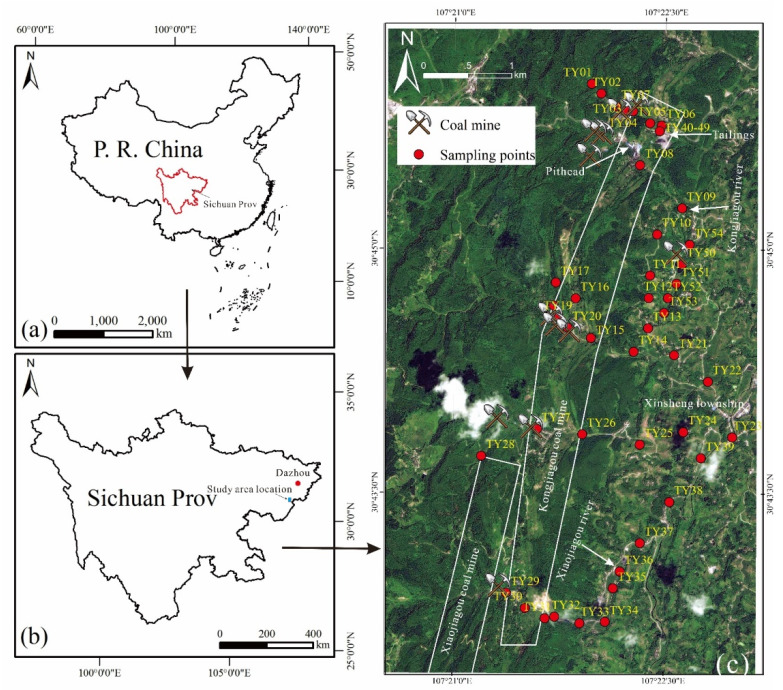
(**a**) The Sichuan Province in China; (**b**) location of study area in Sichuan Province; (**c**) location of soil samples in study area.

The study area is located approximately 15 km northeast of Dazhu County, Dazhou City, Southwest Sichuan Province, China. It has an area of approximately 31.73 km^2^ (107°20′30″–107°23′8″ E, 30°42′22″–30°45′15″ N) and is under the administration of the Xinsheng township of Dazhu County (Figure 1). The surface soil layer is covered with eluvium soil of Quaternary, with a thickness of 0–8 m. The main mineral is quartz, accompanied by typical minerals such as muscovite and calcite. The lithology includes block, gravel silt, and silty clay, which mainly derive from the weathering of coal-bearing sandstone exposed on the surface. The land-use type is farmland, and the farming method is water drought rotation. Sweet potato and rice are planted in the dry and rainy seasons, respectively. The weathered rock in the study area is upper Triassic formation, which is the coal-bearing strata of the area, with a continental sedimentary environment composed of dark gray mudstone, shale, fine sandstone, siltstone, and coal seam. Since the 1960s, more than 10 coal mines have operated in the study area. Except for Kongjiagou coal mine, which is still active, all coal mines were closed between 1990 and 2010 because of the associated pollution and economic changes. The outputs of these coal mines included mainly bright coal, followed by dark coal. Bright coal has a strong shine and a layered structure with thin charcoal. Dark coal has a milder shine, with the formation of lens-shaped specular coal and silk charcoal. The average moisture content in the raw coal is less than 0.9%, the average yield of volatile matter (V_daf_) is 29.04–33.36%, and the CO_2_ content is less than 2% [2].

### 2.2. Soil Sampling and Analysis

On October 2020, 44 surface soil samples and 10 vertical profile soil samples were collected around the coal mine area in Dazhu (Figure 1). The surface soil samples were collected randomly from farmlands on both sides of the road and near the pithead of abandoned coal mines. Three soil cores collected from the top 0–15 cm were combined to produce one composite surface soil sample in the plough layer. A 1 m-deep pit was excavated at the foot of the slope of the tailings, and a vertical profile soil sample was extracted every 10 cm from top to bottom (TY40–TY49). Each sample weighed approximately 1.5–2.0 kg, and the sampling locations were recorded using GPS (Omap). All samples were stored in black polyethylene bags and immediately transported to the laboratory. After being air-dried at room temperature (15 °C) [22]), the samples were homogenized and sieved (<74 μm) for chemical analysis.

X-ray diffraction (XRD) was used for the mineralogical characterization of samples (TY02, TY40, and TY50) in a Rigaku diffractometer (Ultima IV, Akishima-shi, Tokyo, Japan). The conditions were slit fixed at 10 mm, 0.5 mm Pb monochromatic radiation, 40 mA, and 40 kV. The samples were run at a speed of 30°/min (5–80°). A microscope and scanning electron microscope (SEM, Prisma E, Thermo Scientific, Waltham, MA, USA) were used to observe the surface morphology of the soil (TY40) and tailings. The samples were gold-plated in a vacuum environment for elemental analysis by SEM using energy-dispersive X-ray (EDS) detectors.

Soil pH values were measured at a soil: water ratio of 1:2.5 (*w*:*v*) using potentiometry (HJ962-2018) with a pH meter (FE28-Standard, Mettler Toledo, Zurich, Switzerland). Each soil sample was divided into three parts. The first was digested using the method described in (DZ/T 0279-2016) [23]. Approximately 0.1 g of sample was digested in a Teflon crucible using a HCl: HNO_3_: HF: HClO_4_ (2 mL:2 mL:1 mL:1 mL) solution on a hot plate. Subsequently, Fe and Mn were determined using inductively coupled plasma atomic emission spectrometry (ICP–AES) (iCAP 7400, Thermo Fisher Scientific, Waltham, MA, USA), and the concentrations of Ni, Cu, Cd, and Pb were analyzed using inductively coupled plasma mass spectrometry (ICP–MS; Agilent 7700, Agilent Scientific Instruments, Palo Alto, CA, USA). The second part was digested using the method by (HJ491-2019 [24]). Approximately 0.2 g of sample was digested in a digestion tank using a HCl: HNO_3_: HF (3 mL:6 mL:2 mL) solution in a microwave digestion furnace. This method was tested for Cr and Zn using a flame atomic absorption spectrophotometer (GGX-9, Beijing Haiguang Instrument Co., Beijing, China). The third part was digested using the method described in (GB/T 22105-2008) [25]. Approximately 0.2 g of sample was digested in colorimetric tubes using 10 mL aqua regia solution in a boiling water bath for 2 h. Then, 10 mL of preservation solution were added, and As and Hg were tested using an atomic fluorescence photometer (BAF-2000, Beijing Baode Instrument Co., Beijing, China). Two blank samples (one procedure blank and one reagent blank) and two standard samples (GBW07385: GSS-29) were analyzed as duplicates to ensure data reliability. The standard sample was the flood plain sediment of the main river system in China, and it was the standard substance for composition analysis, which was mainly used as the quantity value and quality control standard for sample testing of geological and geochemical investigation and mineral survey. All standard samples were free of pollution and the accuracy of the repeated analysis was less than 5% RSD. The certified and test values of standard samples and the detection limits of each element are presented in Table S1. Metal speciation was extracted sequentially using the BCR sequential extraction procedure [26,27], which includes three sequential extractions and a digestion. The obtained fractions, respectively, included: exchangeable fraction (F1, exchangeable and carbonate-associated fractions), reducible fraction (F2, fraction associated with Fe and Mn oxides), oxidizable fraction (F3, fraction bound to organic matter), and residual fraction (F4).

### 2.3. Evaluation of Soil Contamination

The contamination level of 44 surface soil samples could be analyzed by enrichment factor (EF) and geoaccumulation index (I_geo_).

Enrichment factor (EF) was used to express the enrichment degree of elements in soil, and to judge and evaluate the source of elements in particulate matter [28]. EF can be calculated by [29]:

EF = (C_n_/Fe)_sample_/(C_n_/Fe)_background_, (1)

In this study, where (C_n_/Fe)_sample_ is the ratio of the heavy metals value to the iron concentration in the sample, while (C_n_/Fe)_background_ is the background ratio of the heavy metals value to the iron concentration. EF numerical value can be divided into 5 grades [30]: <2 = minimal pollution; 2–5 = moderate enrichment; 5–20 = significant enrichment; 20–40 = very highly enriched; >40 = extremely enriched.

The metal contamination degree of the soil samples was obtained based on the geoaccumulation index (I_geo_) [31], which was defined by Müller and can be calculated by [32]:

I_geo_ = log_2_[C_n_/1.5B_n_], (2)

where C_n_ is the concentration of metal n in the soil, B_n_ is the background value of metal n, and the factor 1.5 is used to account for possible variations in background data owing to lithological variations. The background values of metals were used as references [33]. The geoaccumulation index can be divided into seven grades [34] and is shown in Table S2.

### 2.4. Statistical Analysis

In the field of environmental science, multivariate analysis has become a more powerful tool than the classical single variable method because it provides an easier means of data analysis [35]. Multivariate analysis methods, such as principal component analysis (PCA) and factor analysis (FA), have been successfully applied to assess soil quality and identify the chemical processes therein [19,36,37,38,39].

To characterize and compare these parameters, the soil properties were analyzed using the SPSS Statistics v22 software (International Business Machines Corporation, Armonk, NY, USA). PCA was applied to the metal variable analysis [40]. Eigenvalues were used to evaluate the number of principal components (PC), known as linear combinations of the old used factors. Factor analysis (FA) was used to determine the common latent structure among variables and reduce PC contribution through further simplification by rotating PCA-defined axes [41]. The Kaiser–Meyer–Olkin (KMO) and Bartlett’s tests of sphericity have been frequently used to test the appropriateness of FA with a correlation coefficient matrix. In total, 9 soil parameters were measured, including Cr, Ni, Cu, Zn, As, Cd, Hg, Pb, and Fe, and these parameters were used in the statistical analysis.

### 2.5. Risk Evaluation

The pollution risk can be quantified and evaluated by the synthesis index [42]. It varies with the obtained metal concentration and the given evaluation criterion for each soil sample [43]. The synthesis index [44] can be calculated as follows:

(3) $$P=\sqrt{\frac{{\left(C_{i}/S_{i}\right)}_{\max}^{2}+(1/n\sum_{i=1}^{n}C_{i}/S_{i})}{2}}$$

where *P* is the synthesis index, *C_i_* is the examined metal concentration for sample *i*, and *S_i_* is the evaluation criterion of the *i*-th kind of metal.

The Soil Environment Quality Risk Control Standard for Soil Contamination of Agricultural Land [45] was adopted as the evaluation criterion. The risk evaluation was performed according to the method mentioned by [46]. First, the synthesis index was calculated for each soil sample based on Equation (3). Second, kriging was evaluated for all soil samples to obtain the total contamination distribution. Finally, the contamination distribution was divided into five grades based on [43,45]. All maps were created using ArcGIS version 10.2 (ESRI, Redlands, CA, USA).

## 3. Results and Discussion

### 3.1. Physical and Chemical Characterization

**Figure 2:**
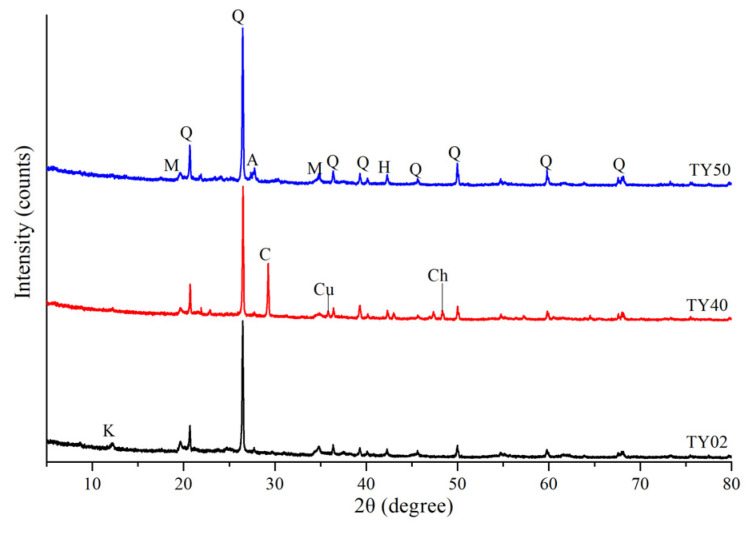
The X-ray diffraction patterns of soils. A—albite; C—calcite; Ch—chalcocite; Cu—cuprite; H—hematite; K—kaolinite; M—muscovite; Q—quartz.

**Figure 3:**
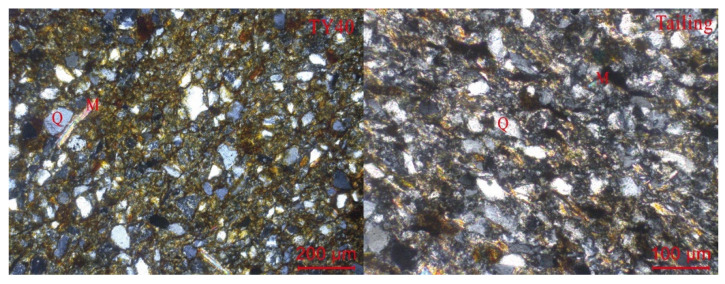
Surface morphology of the soil (TY40) and tailing under microscope. M—muscovite; Q—quartz.

**Figure 4:**
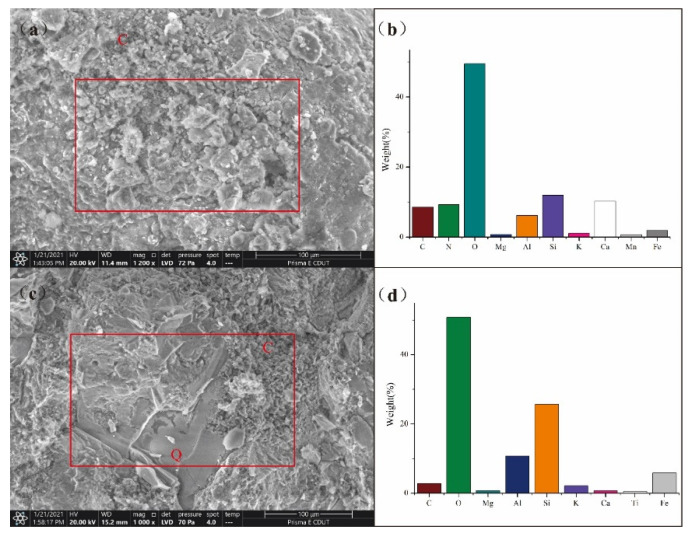
SEM images of the soil (**a**) and tailing (**c**), corresponding EDS spectrum (**b**,**d**). The red box indicates the scope of EDS analysis. Q—quartz; C—clay.

The samples collected in this study were mainly composed of eluvial silty clay of Quaternary. Minerals such as quartz, muscovite, albite, and kaolinite were observed in the soil samples (Figure 2). Hematite was observed in all three samples (TY02, TY40, and TY50) as secondary minerals from oxidation. This may be the reason that hematite occurs in coal and enters the soil during coal mining and transportation. As TY02 and TY40 soil samples were collected near the waste accumulation area, they contained chalcocite and cuprite. Calcite was observed in TY40 and was attributed to the leaching effect of precipitation on tailings, since calcite is found in both tailings and sandstone layers [47]. Both muscovite and quartz were found in TY40 and tailing under the microscope (Figure 3). We observed several of soil samples (TY29, TY34, and TY40) and found that they presented high similarity in the physical phase with the tailing containing coal seam [2]. This indicates that the main components of farmland soil originate from the minerals derived from sandstone weathering. In addition, carbonate minerals and siliceous rocks were observed in quartz fractures as interstitial materials (Figure 3). The SEM images show that the surfaces of soil and tailing samples were irregular (Figure 4a,c). From the figure, quartz could be observed, and pores were filled with weathered fragmental material. Figure 4b shows the EDS analysis results. The main elements in the soil were C, N, O, Si, and Ca, followed by metal elements such as Fe, Mn, Al, and Mg. This indicates that the surfaces of quartz and carbonate minerals were covered by Fe-Mn oxide. (Figure 4d) shows the EDS analysis result of tailing. The main elements were O, Si, Al, and Fe, which indicates that the sandstone layer contained metal elements such as Fe, which remain in the soil in the form of oxides after weathering. These results illustrate the environmental impact of intense mining activities on soil quality.

### 3.2. Concentration and Speciation of Heavy Metals

The descriptive statistical results of pH and metal concentration of soil samples are shown in Table 1 and the concentrations of these elements are listed in Table S3. The concentrations of the 10 investigated metals varied widely. Most soil samples were weakly alkaline, and their average pH was 7.22. Fe was the most abundant metal, with a mean concentration of 3.55 wt%. The mean value of Mn was 956.27 mg/kg, and that of Zn was 89.07 mg/kg. The average concentrations of Ni, Cu, and Pb were relatively similar, at 34.85, 26.38, and 28.47 mg/kg, respectively. The mean Cr and As concentrations were 58.19 and 8.51 mg/kg, respectively. The concentrations of Cd and Hg were significantly lower than those of other metals, at 0.41 and 0.14 mg/kg, respectively. Except for As and Cr, the concentrations of most metals exceeded their respective background values [33], especially those of Cd and Hg, the average concentrations of which were four and two times the background values, respectively. In the surface soil, increased concentrations of heavy metals were attributed to the dual function of secondary enrichment and parent rock inheritance. Ni and Mn presented high coefficients of variation (CV), at 194% and 218%, respectively, which exceeded 100%. Therefore, they presented a greater variation than other metals, and their high contents were strongly associated with a wide range of human activities. The CV values of Zn, As, and Cd were 52%, 54%, and 85%, respectively, which indicate a high level of spatial variation (CV > 50%) [48]. Similar to the results of most researchers [49,50], the spatial variation of coal mine pits resulted in metal data heterogeneity. The kurtosis values of Ni, Zn, Cd, Pb, and Mn were higher than 10, which indicated that there was great heterogeneity in the distribution of these elements in the soil [51]. In addition, the skewness values of all elements were greater than 0, which indicated that most metal concentrations were at relatively low values.

**Figure 5:**
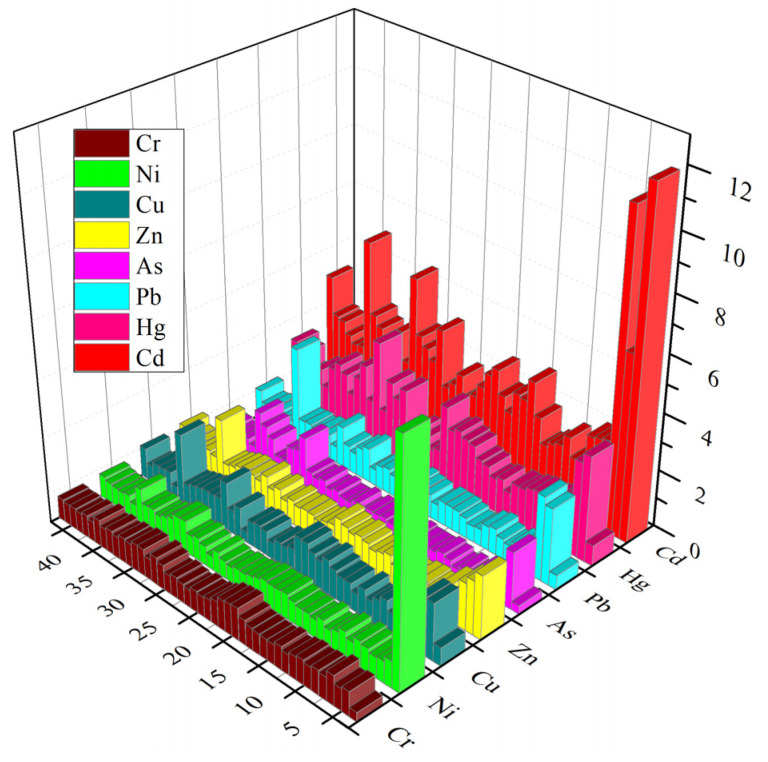
Three-dimensional plots of enrichment factor (EF).

The EF index values are presented in Figure 5 and in Table S4. According to the EF index values, heavy metals showed a wide range of enrichment at each sampling location. Cd had the highest degree of enrichment, belonging to moderate enrichment and significant enrichment. The Hg and Pb enrichment level of most soil samples was minimal pollution, but a small part showed significant enrichment. The lower enrichment degree was found for Cr and Ni, except in TY01. Briefly, the order of the average values of the EF index was Cd > Hg > Pb > As > Zn > Cu > Ni > Cr. The high contents of Fe/Al/Mn oxides, carbonaceous species, and clay (Al_2_/SiO_2_) were closely related to the enrichment of heavy metals in the soil. Human activities such as coal combustion, waste incineration, and transportation can release a large amount of dust containing Cd and Hg into the atmosphere, which is then enriched through natural sedimentation and rain [52]. In addition, the heavy application of chemical fertilizers and pesticides in agricultural production can lead to Cd pollution [53]. These results illustrate the effects of weathering of primary minerals for soil formation and the impacts of mining activities on the concentrations of heavy metals in the soil of the mining area.

**Figure 6:**
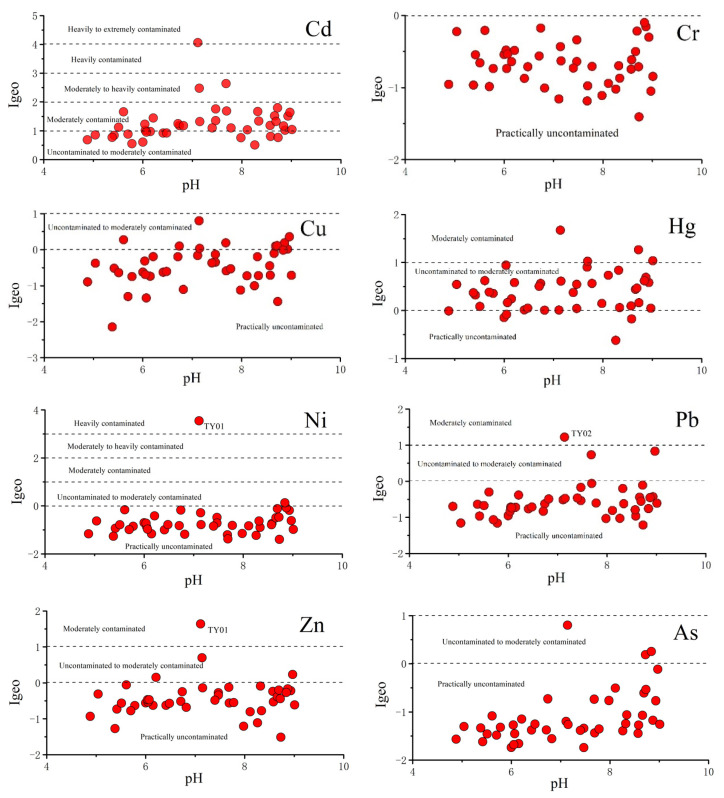
The index of geoaccumulation (I_geo_) of soils in the study area.

The mean Cd concentration exceeded the standard value given in [45] by 36% (Table 1). The results of the geoaccumulation index (I_geo_) are shown in Figure 6 and listed in Table S5. Moreover, Figure 6 indicated that soils in the coal mine area were significantly polluted by Cd, which presented Class 5 (heavily to extremely contaminated, TY01) and Class 3 (moderately–heavily contaminated, TY02 and TY03). In addition, the I_geo_ of Cd in most soil samples were classified as Class 2 (moderately contaminated). Hg, Pb, and Zn presented lower I_geo_ values, dominated by Classes 2 (moderately contaminated) and 1 (uncontaminated–moderately contaminated). Because the TY01 and TY02 mixed soil samples were collected near the pithead of an abandoned coal mine, they were more strongly affected by the deposition of primary minerals and immersional wetting of tailings, which resulted in extremely high I_geo_ values for Ni, Pb, and Zn. In addition, As and Cu were associated with I_geo_ Classes 1 and 0 (practically uncontaminated). Cr presented the lowest pollution, and it was the only contaminant with I_geo_ Class 0 for all samples. According to the I_geo_ results, heavy metal pollution in the study area was at a safe level, except for Hg and Cd. The main pollution sources are likely the tailings, dust piled up in mining areas, and settlement of industrial coal. The high concentration of Cd caused heavy metal pollution in the agricultural soils of the study area. Accordingly, long-term consumption of rice, vegetables, fruits, and water seriously polluted by Cd are likely to lead to chronic poisoning [54,55]. In addition, Cd has become the most serious heavy metal soil pollutant in China and substantially affects the quality and yield of crops [4,56].

**Figure 7:**
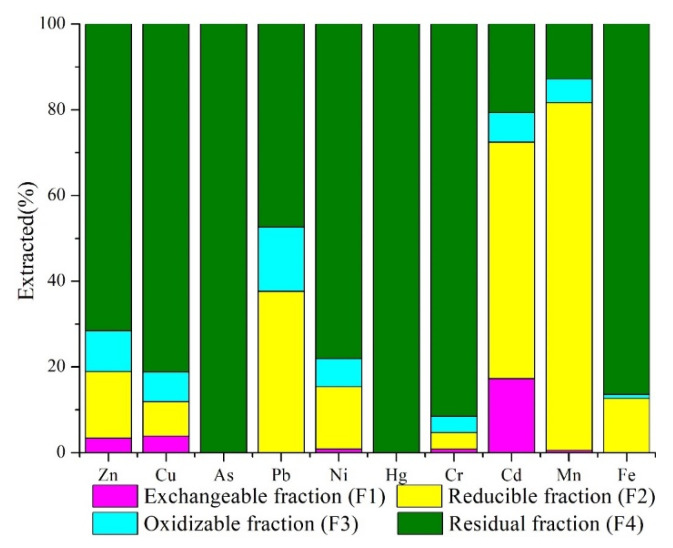
The percentages of metals chemical speciation in soils.

Figure 7 shows the chemical speciation percentages of the metals in the soil sample (TY40), which was at the foot of the slope of the tailings. In addition, a large number of crops were planted near the site from which this soil was sampled. In general, the residual fraction was dominant in most metals (F4), except for Cd and Mn, in which the reducible fraction was dominant (F2) (Cd: 55.17%; Mn: 81.16%). Owing to their detection limit, only residual fractions were observed for As and Hg (100%). Cd yielded the highest exchangeable fraction (F1), up to 17.24%, whereas the exchangeable fraction of As, Pb, Hg, and Fe were not detected. Pb presented the highest oxidizable fraction (F3) (14.98%).

The percentages of different Cd fractions for F1, F2, F3, and F4 were 17.24%, 55.17%, 6.9%, and 20.69%, respectively. The high proportion exchangeable fraction of Cd indicated that its bioavailability was high, and its migration ability in the soil was strong [57]. In addition, the release of exchangeable elements can cause a large amount of cation replacement in soil through precipitation (acid rain), which leads to substantial nutrient deficiency and toxicity to plants [58]. The reducible fraction generally exists in the outer capsule of minerals and fine powder particles, with strong exclusive adsorption, and it is easily released when the redox potential of the water body decreases or the water lacks oxygen [59]. Both exchangeable and reducible fractions of Cd showed great bioavailability, which indicated that Cd presented a greater pollution to the environment than other elements in the study area.

The proportions of reducible and residual fractions of Pb were similar, at 37.65% and 47.37%, respectively. Generally, the release of Pb in reducible species is difficult, but under anoxic conditions, generated by a soil water saturation (for example) with no water transfer, Pb will be released, thereby causing secondary pollution [60]. In addition, the proportions of oxidizable fraction of Pb was 14.98%, which indicated that Pb easily formed complexes or chelates with humic acid and other organic matter in sediments, and it then coprecipitated with sulfide. The oxidable fraction of heavy metals reflects the aquatic activities and effects of the discharge of organic-rich sewage. Heavy metals in this form are relatively stable in the soil, but under strong oxidation conditions, their mobility can increase, and they can enter the water [61].

The high ratio of residual fraction in As, Hg, Cr, and Fe indicated a strong combination with the crystal structure of minerals, which was stable under natural conditions with a low transferability [62]. Because these elements mainly existed in the crystal lattice of the minerals, a non-anthropogenic source was suggested for the metals in the residual fraction [63]. Similar to the conclusion of many researchers [64], Cu and Zn appeared mostly in the residual and reducible fractions. In this study, soil particles were mainly composed of silty clay, and the adsorption of heavy metals increased with the decrease in particle size [65]. Mn is mainly found in the reducible fraction (from Fe-Mn oxides) and less in the oxidizable fraction (bound to OM) [66]. Many studies have shown that Ni can preferentially combine with aluminosilicate minerals (e.g., kaolinite and muscovite (Figure 2) [67], resulting in its residual fraction being high (78.06%).

### 3.3. Statistical Analysis

To further investigate the relationship between heavy metals in surface soils, the Pearson correlation coefficients were calculated, and the results are shown in Table 2. Ni was positively (*p* < 0.01) correlated with Zn, Cd, and Fe, whereas Cu was positively correlated with Cr, Zn, As, Hg, and Pb. In addition, Zn exhibited positive significant correlations with Cd, Pb, and Fe.

In this study, the sphericity (0.6) was larger than 0.5, and the KMO result was less than 0.001, which indicated that the data was suitable for FA [68]. Based on eigenvalues (eigenvalue > 1), three main factors explained 87.918% of the total variance. The variance contribution rate of Factor 1 (F1) was 43.106%, and was positively correlated with Ni, Cd, Zn, and Fe (0.975, 0.964, 0.934, and 0.589), respectively (Table 3). The interrelationships between Ni, Zn, and Cd suggest the influence of local human activities (domestic waste and fertilization) and lithology (weathering of parent rocks) on soil samples [41]. F2, which explained 27.906% of the total variance, was highly positively correlated with Pb, As, Hg, and Cu (0.885, 0.856, 0.831, and 0.8), respectively. Many researchers [69,70] believe that the main reason for the enrichment of Cu and As in soils is the application of chemical fertilizers and pesticides. The high correlation between Hg and Pb was attributed to the automobile emissions from coal transportation and the fly ash produced by coal combustion [71]). F3 presented a variance contribution rate of 16.906%, and Cr and Fe presented high loads (0.954 and 0.734, respectively). The high ratio of residual fraction in Cr and Fe indicated a high correlation with soil-forming parent material. Therefore, the analysis indicates that F1 represents anthropogenic and lithologic sources, which are affected by mining activities; F2 represents anthropogenic sources, such as fertilizers and traffic pollution; and F3 represents the contribution of metals from soil-forming parent material.

### 3.4. Spatial Distribution and Risk Assessment

**Figure 8:**
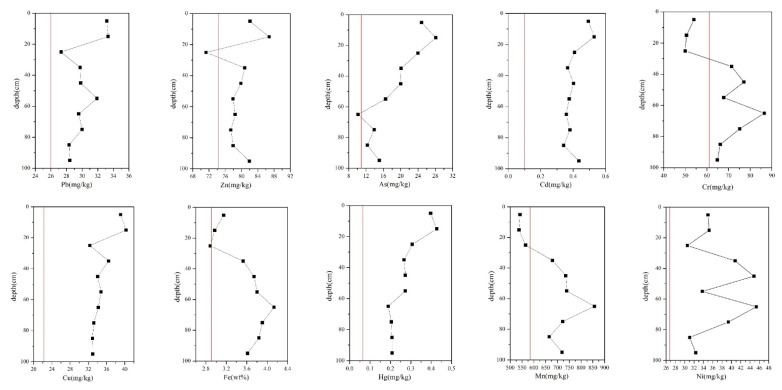
Distribution of metal concentrations in vertical profile soils under the tailings. The red line indicates background value.

Figure 8 shows the distribution of metal concentrations in the vertical profile of the soils under the tailings. The mean concentrations of all metals exceeded the background values [33], especially those of Cd and Hg, which were four times the background values. Only the concentration of Cd exceeded the limit value [45]. The metal concentrations in the vertical profile of the soils (TY40–49) are listed in Table S3.

In general, the concentrations of most metals decreased with depth, which indicated that most metals in the farmland were still in the plow layer. The average deviation of Mn was the highest (79.1°), followed by Cr (9.2°). The average deviations of other metals were found to be within ±5%. In Section 3.1, calcite and cuprite were found in TY40, which was sampled from the surface of the deep pit at the foot of the slope of the tailings. This may be because the atmospheric precipitation would flow through the tailings before entering the soil, bringing these minerals in the tailings into the surface soil. Therefore, heavy metals were easier to enrich in surface layer. Fe and Mn concentrations were lower at 0–20 cm, and higher at 60–80 cm, which was attributed to their predominant reducible (F2) and residual fractions (F4). The weak acid water produced by tailing leaching reacted with Fe and Mn oxides in the surface layer, which led to their enrichment in deeper soil layers. The Cr concentration was low in the surface soil but rich at 70 cm. With the pH increase (mean pH of 8.8), the Cr adsorption on soil was clearly weakened. This occurred because higher pH values lead to more negative charges on the soil surface. This increases the probability of the formation of complexes with organic acids, which decrease the adsorption capacity and enhance the mobility of heavy metals in the soil [72]. In this study, the surface–bottom soil pH showed a decreasing trend (from 9.05 to 8.54), which led to more Cr being adsorbed on the clay in a deeper position than on the surface. The vertical distribution of Ni concentration in the soil first decreased and then increased. The highest concentration of Ni was at 70 cm. When the permeability of shallow soil was good, the density of deep soil was high, the water retention was good, and the ability of Ni for downward migration would increase [73]. In addition, the accumulation of Pb was likely related to the transportation of leaded gasoline [67].

**Figure 9:**
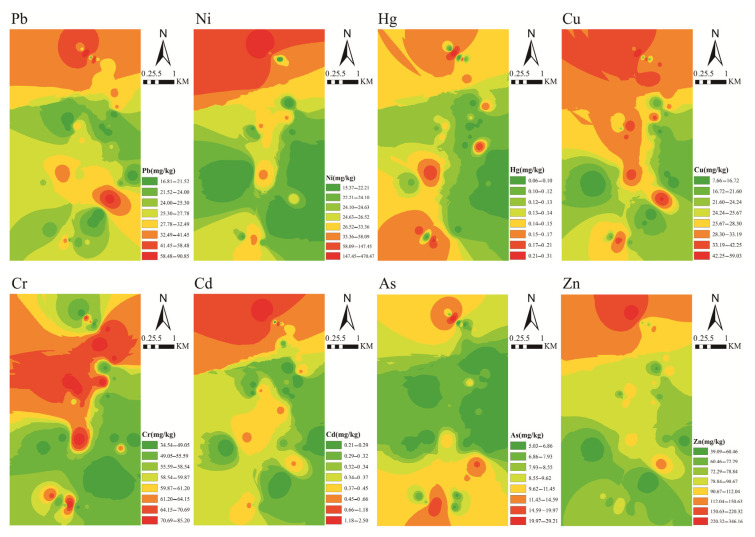
Spatial distributions of metals in farmland soils.

The spatial analysis of metals in the soils of the study area are shown in Figure 9, with a clear spatial distribution pattern of heavy metals in the soil. Pb was mainly distributed in the north and middle parts of the study area. The highest Pb values were observed for TY02 (91.01 mg/kg), which was close to the entrance and exit of the transportation center. This suggested that industrial activities substantially affected the enrichment of Pb in the soil. The concentrations of Ni and Zn presented similar spatial distribution patterns. High concentrations of Ni and Zn were mainly observed in areas where human activities were concentrated, and they were observed throughout the entire study area, from north to south. The high Ni and Zn contents in these other areas were mainly caused by geochemical-related industrial activities, such as application of pesticides and chemical fertilizers. The spatial distribution of Cu concentration was similar to those of Ni and Zn, but it was more intense, which indicated that human activities had a more severe impact on Cu.

The spatial distribution patterns of Cr were higher in the north and lower in the south. High Cd concentration was mainly distributed in the north of the study area, where pulverized fuel ash pipelines and tailings were located. A large number of pesticides and chemical additives are used in agricultural activities both north and south of the study area, resulting in the enrichment of As in the soils. High Hg concentrations were observed southwest of the Xiaojiagou coal mine, which characterized a decrease in spatial distribution from northeast to southwest. The Xiaojiagou coal mine, located south of the study area, had been closed, whereas the Kongjiagou coal mine in the north was still operative, so the northern road was the main coal transportation road. Moreover, there was a large number of coal companies and tailings in the north of the research area, which likely justified the enrichment of Zn, Ni, Cr, Cu, and Cd in the northern region. This also showed that the mining operations exerted a more severe impact on the environment, whereas the closure of mines was conducive to the recovery of the local environment.

**Figure 10:**
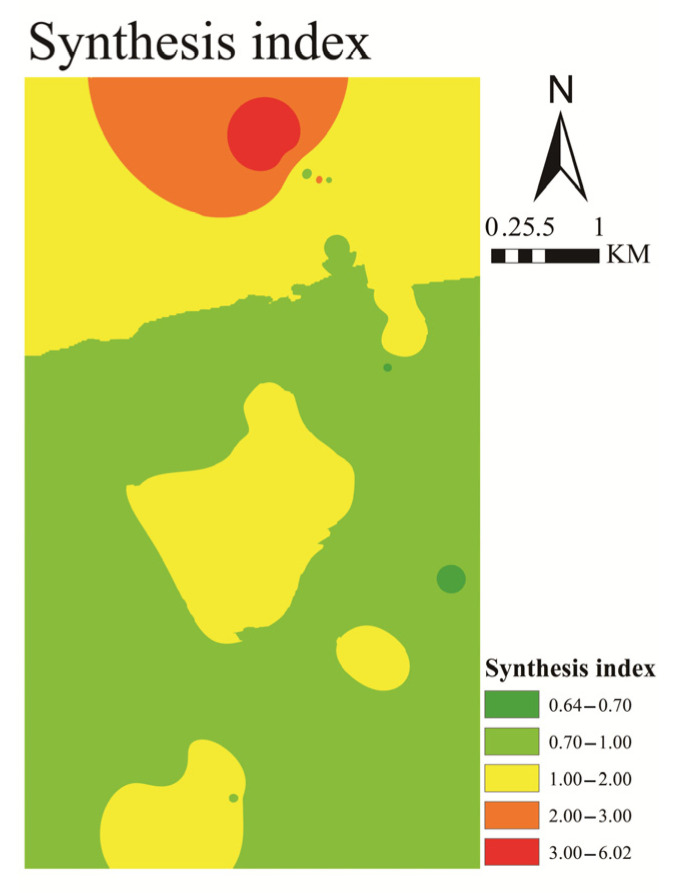
The synthesis index map of metals in study area.

We conducted a risk assessment for the study area to provide a basis for effective suggestions for policy makers and farmers. The synthesis index was adopted for the assessment. The results are shown in Table 4 and Figure 10, and they indicate that there were few safe areas, accounting for only 0.06 km^2^ (0.21%). This was mainly attributed to the concentration of Cd, which exceeded the limit value in most areas. Most of the soil (56.90%, 18.06 km^2^) belonged to guard grade (0.7 < *p* ≤ 1), including central and southern parts of the study area, where human activities were concentrated. Owing to the use of chemical fertilizers and pesticides, these concentrated areas belonged to low pollution grade. Considering the pithead of Kongjiagou coal mine as the boundary, several factors in the northern area caused them to belong to Levels 3–5, including coal transportation, tailings accumulation, and chemical plants. These areas were classified under moderate (1.96 km^2^, 6.17%) and severe (0.36 km^2^, 1.13%) with high concentrations of Cd (2.51 mg/kg) and Pb (91.01 mg/kg). Some of the physiological effects of chronic exposure to waterborne cadmium at sub-lethal concentrations are manifested in the form of reduction in growth and changes in hematology and enzyme activity [74]. Lead at sub-lethal levels destroys normal metabolic processes by disrupting calcium and sodium homeostasis [75]. In view of such risks, protective measures are required to avoid heavy metal contamination of vegetables and ensure food safety for human consumption. The risk assessment results can be used as a basis to estimate the environmental cleaning costs of coal mine areas. The classification suggests that vegetables should not be planted in high-risk areas, whereas in low-risk areas, the cultivation of crops should consider the economic and environmental impacts.

## 4. Conclusions

In this study, we investigated the mineralogical characterization, concentrations, speciation, statistical analysis, spatial distribution, and risk assessment of metals in a coal mine area in Dazhu, China. Except for As and Cr, the concentrations of all metals (Fe, Ni, Cu, Zn, Mn, Cd, Hg, and Pb) exceeded the background values [33], which indicated that the weathering of primary minerals in soil formation and coal mining activities substantially affected soil quality. Cd pollution was the most intense, and it exceeded the limit by 36% [45]. Cd was classified under I_geo_ Class 5 (heavily–extremely contaminated) or Class 6 (extremely contaminated). The XRD and SEM analyses indicated the presence of many secondary minerals, which likely influenced the concentrations of heavy metals. The residual fraction was dominant for most metals, except Cd and Mn, for which the reducible fraction was dominant (Cd: 55.17%; Mn: 81.16%). The concentrations of most metals decreased with depth, which indicated that most metals in the farmlands originated from human activities and remained in the plow layer. The statistical analysis showed that Factor 1 can represent anthropogenic and lithologic sources, which are affected by mining activities, whereas Factor 2 can represent anthropogenic sources such as fertilizers and traffic pollution. Factor 3 represented the contribution of metals from soil-forming parent material. The heavy metal concentration in the mining area was high and presented high risk, so the area is not suitable for agriculture. The mining activities had a severe impact on the environment, whereas areas with closed mines were associated with the recovery of the local environment.

## Figures and Tables

**Table 1:** pH and concentration of metals in soil samples of coal mine area.

Item	pH	Cr	Ni	Cu	Zn	As	Cd	Hg	Pb	Mn	Fe
		mg/kg	mg/kg	mg/kg	mg/kg	mg/kg	mg/kg	mg/kg	mg/kg	mg/kg	wt%
N ^a^	44	44	44	44	44	44	44	44	44	44	44
Minimum ^a^	4.88	34.52	15.36	7.66	39.04	5.03	0.21	0.06	16.80	258.39	2.19
Maximum ^a^	9.01	85.37	471.61	59.11	346.68	29.27	2.51	0.31	91.01	14314.45	6.18
Mean ^a^	7.22	58.19	34.85	26.38	89.07	8.51	0.41	0.14	28.47	956.27	3.55
Median ^a^	7.28	56.20	23.39	24.26	78.77	6.96	0.33	0.13	25.15	622.01	3.49
25th ^a^	6.07	47.45	20.43	20.52	72.10	6.20	0.28	0.1	22.16	460.06	3
75th ^a^	8.4	64.81	28.72	32.05	94.81	8.51	0.41	0.15	28.51	780.32	3.99
Skewness ^a^	−0.14	0.45	6.53	0.87	4.27	2.94	5.21	1.74	3.14	6.44	0.84
Kurtosis ^a^	−1.32	−0.43	43.10	1.66	22.64	9.89	30.43	4.84	10.75	42.2	1.55
SD ^a^	1.27	12.51	67.70	9.72	46.44	4.60	0.35	0.04	13.99	2080.77	0.8
CV ^a^	0.18	0.22	1.94	0.37	0.52	0.54	0.85	0.33	0.49	2.18	0.22
TY40-49 ^b^	8.8	66.3	36.81	35.02	79.43	18.48	0.41	0.28	30.15	676.18	3.56
Background values ^c^	-	61	26.9	22.60	74.2	11.2	0.1	0.065	26	583	2.94
Limit value ^d^	-	200	100	100	250	30	0.3	2.4	120	-	-

^a^ Item of surface soil. ^b^ Item of profile soil. ^c^ Background values for soils in China [33]. ^d^ Soil Environment Quality Risk Control Standard for Soil Contamination of Agricultural Land [45].

**Table 2:** The correlation matrix of metals and pH in soil.

	Cr	Ni	Cu	Zn	As	Cd	Hg	Pb	Fe
Cr	1								
Ni	−0.128	1							
Cu	0.506 **	0.132	1						
Zn	0.055	0.882 **	0.511 **	1					
As	0.166	0.007	0.633 **	0.259	1				
Cd	−0.183	0.921 **	0.340 *	0.927 **	0.18	1			
Hg	0.251	−0.094	0.604 **	0.186	0.636 **	0.143	1		
Pb	−0.031	0.005	0.727 **	0.389 **	0.659 **	0.326 *	0.596 **	1	
Fe	0.543 **	0.568 **	0.331 *	0.606 **	−0.022	0.473 **	−0.025	−0.05	1

* Correlation is significant at the 0.05 level; ** Correlation is significant at the 0.01 level.

**Table 3:** Factor loadings in soils.

	F1	F2	F3	Communalities
Ni	**0.975**	−0.102	0.019	0.962
Cd	**0.964**	0.192	−0.084	0.962
Zn	**0.934**	0.275	0.146	0.879
Pb	0.167	**0.885**	−0.147	0.969
As	0.043	**0.856**	0.024	0.735
Hg	−0.047	**0.831**	0.112	0.972
Cu	0.233	**0.8**	0.43	0.705
Cr	−0.152	0.169	**0.954**	0.832
Fe	**0.589**	−0.099	**0.734**	0.896
Eigenvalues	0.975	−0.102	0.019	
% of variance explained	43.106	27.906	16.906	
Cumulative % of variance	43.106	71.012	87.918	

Loading values for the PC axis higher than +0.5 and lower than −0.5 are given in bold.

**Table 4:** The evaluation standard and results in study area.

Level	*p* ^a^	Grade	Area (km^2^)	Percent (%)
1	*p* ≤ 0.7	Safety	0.06	0.21
2	0.7 < ∂*p* ≤ 1	Guard	18.06	56.90
3	1 < *p* ≤ 2	Low pollution	11.29	35.59
4	2 < *p* ≤ 3	Moderate pollution	1.96	6.17
5	*p* < 3	Severe pollution	0.36	1.13

^a^ The standard based on [43,45].

## Data Availability

Not applicable.

## Supplementary Materials

The following supporting information can be downloaded at: https://www.mdpi.com/article/10.3390/ijerph19116493/s1, Table S1: The certified and test values of standard samples and the detection limits of each element, Table S2: Six classes of the geoaccumulation index, Table S3: pH and concentrations of soil samples, Table S4: The EF index values, Table S5: The geoaccumulation index (I_geo_).
