# Supplementary material for: Pollution Characteristics and Spatial Distribution of Heavy Metals in Coal-Bearing Sandstone Soil: A Case Study of Coal Mine Area in Southwest China
Source: Int J Environ Res Public Health. 2022 May 26;19(11):6493. doi: 10.3390/ijerph19116493 (PMC9180046; doi:10.3390/ijerph19116493)
Supplement: Supplementary file 1 [file ijerph-19-06493-s001.zip › ijerph-1723519-supplementary.pdf]

Table S1. The certified and test values of standard samples and the detection limits of each element.

	Cr	Ni	Cu	Zn	As	Cd	Hg	Pb	Mn	TFe_2O_3
	mg/kg	mg/kg	mg/kg	mg/kg	mg/kg	mg/kg	mg/kg	mg/kg	mg/kg	wt%
Certified value	80 ± 5	38 ± 2	35 ± 2	96 ± 4	9.3 ± 0.8	0.28 ± 0.02	0.15 ± 0.02	32 ± 3	760 ± 16	5.44 ± 0.15
Test value	78.18	37.06	34.11	93.25	9.46	0.29	0.15	33.77	770.53	5.38
Detection limits	4	0.2	0.5	10	0.01	0.021	0.002	0.7	0.02	0.0009

Table S2. Six classes of the geoaccumulation index.

Class	Value	Soil Dust Quality
0	$I_{geo} \leq 0$	Practically uncontaminated
1	$0 < I_{geo} < 1$	Uncontaminated to moderately contaminated
2	$1 < I_{geo} < 2$	Moderately contaminated
3	$2 < I_{geo} < 3$	Moderately to heavily contaminated
4	$3 < I_{geo} < 4$	Heavily contaminated
5	$4 < I_{geo} < 5$	Heavily to extremely contaminated
6	$5 < I_{geo}$	Extremely contaminated

Table S3. pH and concentrations of soil samples.

No	pH	Cr	Ni	Cu	Zn	As	Cd	Hg	Pb	Mn	Fe
		mg/kg	mg/kg	mg/kg	mg/kg	mg/kg	mg/kg	mg/kg	mg/kg	mg/kg	wt%
TY01	7.11	40.97	471.61	30.33	346.68	7.34	2.51	0.10	27.29	14314.45	6.18
TY02	7.14	67.76	33.06	59.11	180.88	29.27	0.83	0.31	91.01	476.74	3.83
TY03	7.68	40.19	17.52	38.64	102.27	10.08	0.93	0.18	64.95	650.22	2.49
TY04	6.15	58.67	17.99	20.33	72.19	5.32	0.30	0.12	23.63	402.75	2.71
TY05	6.21	65.33	30.20	29.69	123.78	7.59	0.41	0.15	29.87	832.18	3.89
TY06	8.93	74.16	35.49	34.21	95.71	9.86	0.43	0.15	28.97	963.19	4.16
TY07	6.00	62.81	24.80	22.01	75.66	5.03	0.23	0.09	20.12	649.00	3.78
TY08	8.87	82.09	38.25	38.63	99.05	7.45	0.31	0.16	28.46	777.59	4.56
TY09	7.47	72.34	28.96	30.93	92.02	5.03	0.38	0.14	34.66	729.07	4.33
TY10	4.88	47.15	18.02	18.24	58.36	5.67	0.24	0.10	24.04	439.14	3.22
TY11	6.74	80.97	35.79	36.35	93.88	10.14	0.34	0.14	25.23	788.51	4.80
TY12	8.58	59.75	24.25	20.72	77.15	6.96	0.26	0.09	19.99	549.53	4.08
TY13	8.57	54.55	23.40	24.82	94.51	6.18	0.34	0.10	22.50	719.36	3.63
TY14	7.47	58.68	24.56	26.63	87.87	6.64	0.51	0.10	26.84	837.03	3.84
TY15	8.66	64.63	28.64	31.54	85.13	8.02	0.43	0.13	28.64	911.03	4.05
TY16	5.61	79.18	36.05	41.02	107.09	7.94	0.48	0.15	31.65	986.24	4.36
TY17	5.04	78.30	26.10	26.08	89.67	6.81	0.27	0.14	17.45	258.39	3.33
TY18	5.42	62.62	21.17	23.69	67.04	5.47	0.27	0.12	19.96	274.16	2.74
TY19	6.71	61.94	22.84	29.61	77.97	6.49	0.36	0.14	21.90	339.66	2.85
TY20	7.15	59.09	23.45	34.75	101.12	7.02	0.38	0.15	28.03	467.04	3.29
TY21	9.01	50.88	20.45	20.68	72.79	7.04	0.31	0.20	25.52	616.25	3.25
TY22	6.05	54.90	22.04	20.97	76.83	5.25	0.30	0.09	23.73	679.33	3.80
TY23	8.26	45.06	17.23	16.92	51.57	6.41	0.21	0.06	19.08	573.79	2.93
TY24	6.82	45.49	17.80	15.76	69.50	5.72	0.34	0.10	27.77	575.00	3.54
TY25	7.40	55.10	22.46	26.25	79.57	6.44	0.32	0.13	28.29	807.92	3.97
TY26	8.69	78.68	37.23	36.30	96.89	11.02	0.38	0.14	26.52	940.14	5.17
TY27	7.69	46.48	15.58	22.52	75.53	6.21	0.48	0.20	37.39	1504.23	3.48

TY28	5.38	46.83	16.77	7.66	46.06	6.68	0.26	0.13	25.06	334.81	2.66
TY29	6.04	65.55	24.50	27.23	80.44	6.97	0.35	0.19	21.88	363.93	3.26
TY30	8.34	50.02	21.71	20.54	65.03	8.06	0.38	0.10	25.34	656.28	3.18
TY31	8.72	55.84	29.12	36.60	82.06	19.14	0.52	0.24	36.14	627.77	2.81
TY32	8.84	85.37	44.15	33.57	92.37	20.05	0.34	0.15	23.05	581.74	4.33
TY33	8.11	47.55	22.70	20.48	63.73	11.82	0.31	0.16	22.25	472.92	2.72
TY34	7.78	56.04	23.00	23.46	76.13	6.58	0.32	0.14	25.62	316.68	3.09
TY35	6.41	49.93	20.22	21.94	72.20	6.47	0.28	0.10	22.93	397.59	3.15
TY36	7.98	42.33	18.16	15.57	48.16	9.89	0.25	0.11	19.05	424.10	2.43
TY37	8.73	34.52	15.36	12.49	39.04	11.60	0.26	0.11	16.80	537.09	2.19
TY38	8.97	44.15	26.38	43.48	130.92	15.50	0.47	0.10	69.38	1729.86	2.31
TY39	6.07	63.04	20.62	13.38	80.47	6.14	0.29	0.11	23.42	735.19	4.25
TY40	9.05	64.75	32.34	32.97	81.92	15.06	0.44	0.21	28.43	719.85	3.62
TY41	9.00	66.17	31.04	32.90	77.94	12.28	0.34	0.21	28.31	665.44	3.84
TY42	8.89	75.09	39.33	33.19	77.37	13.86	0.38	0.20	29.99	722.64	3.90
TY43	8.88	86.43	45.34	34.20	78.43	10.16	0.36	0.19	29.55	856.56	4.13
TY44	8.70	67.77	33.76	34.79	77.88	16.52	0.38	0.27	31.90	739.38	3.80
TY45	8.71	77.06	44.80	34.05	79.84	19.97	0.40	0.27	29.82	735.19	3.74
TY46	8.73	71.38	40.77	36.44	80.79	20.07	0.37	0.27	29.75	679.39	3.53
TY47	8.74	49.87	30.56	32.32	71.27	23.98	0.41	0.31	27.31	565.00	2.88
TY48	8.54	50.50	35.22	40.27	86.78	28.06	0.53	0.43	33.33	537.09	2.97
TY49	8.72	53.97	34.97	39.07	82.10	24.82	0.49	0.40	33.15	541.28	3.15
TY50	5.78	54.91	22.42	20.20	71.85	6.74	0.22	0.13	17.44	629.17	3.33
TY51	6.48	55.83	23.49	22.29	75.01	7.06	0.29	0.10	23.74	599.87	3.81
TY52	5.51	58.00	23.38	21.76	75.34	6.12	0.33	0.10	24.39	334.81	3.71
TY53	5.70	46.15	20.37	13.77	65.00	6.02	0.28	0.13	18.61	503.61	3.03
TY54	8.32	56.36	26.15	29.62	104.60	7.11	0.48	0.18	33.92	768.67	3.50

Table S4. The EF index values.

No	Cr	Ni	Cu	Zn	As	Cd	Hg	Pb
TY01	0.32	8.34	0.64	2.22	0.31	11.93	0.72	0.50
TY02	0.85	0.94	2.01	1.87	2.01	6.41	3.69	2.69
TY03	0.78	0.77	2.02	1.63	1.06	11.04	3.34	2.95
TY04	1.04	0.72	0.97	1.05	0.52	3.20	1.94	0.98
TY05	0.81	0.85	0.99	1.26	0.51	3.10	1.71	0.87
TY06	0.86	0.93	1.07	0.91	0.62	3.03	1.60	0.79
TY07	0.80	0.72	0.76	0.79	0.35	1.78	1.06	0.60
TY08	0.87	0.92	1.10	0.86	0.43	1.97	1.57	0.71
TY09	0.81	0.73	0.93	0.84	0.31	2.61	1.50	0.91
TY10	0.71	0.61	0.74	0.72	0.46	2.21	1.37	0.84
TY11	0.81	0.81	0.98	0.77	0.55	2.09	1.37	0.59
TY12	0.71	0.65	0.66	0.75	0.45	1.89	0.96	0.55
TY13	0.72	0.70	0.89	1.03	0.45	2.77	1.30	0.70
TY14	0.74	0.70	0.90	0.91	0.45	3.91	1.19	0.79
TY15	0.77	0.77	1.01	0.83	0.52	3.13	1.49	0.80
TY16	0.88	0.90	1.22	0.97	0.48	3.21	1.57	0.82
TY17	1.13	0.86	1.02	1.07	0.54	2.41	1.94	0.59
TY18	1.10	0.84	1.12	0.97	0.52	2.89	2.02	0.82
TY19	1.05	0.87	1.35	1.08	0.60	3.68	2.20	0.87

TY20	0.87	0.78	1.37	1.22	0.56	3.36	2.06	0.96
TY21	0.75	0.69	0.83	0.89	0.57	2.80	2.80	0.89
TY22	0.70	0.63	0.72	0.80	0.36	2.35	1.10	0.71
TY23	0.74	0.64	0.75	0.70	0.57	2.15	0.99	0.74
TY24	0.62	0.55	0.58	0.78	0.42	2.82	1.26	0.89
TY25	0.67	0.62	0.86	0.79	0.43	2.39	1.45	0.81
TY26	0.73	0.79	0.91	0.74	0.56	2.14	1.19	0.58
TY27	0.64	0.49	0.84	0.86	0.47	4.10	2.60	1.22
TY28	0.85	0.69	0.38	0.69	0.66	2.84	2.16	1.07
TY29	0.97	0.82	1.09	0.98	0.56	3.18	2.62	0.76
TY30	0.76	0.75	0.84	0.81	0.67	3.52	1.46	0.90
TY31	0.96	1.13	1.70	1.16	1.79	5.47	3.80	1.46
TY32	0.95	1.11	1.01	0.85	1.22	2.30	1.57	0.60
TY33	0.84	0.91	0.98	0.93	1.14	3.33	2.71	0.92
TY34	0.87	0.81	0.99	0.98	0.56	3.07	2.12	0.94
TY35	0.76	0.70	0.91	0.91	0.54	2.66	1.42	0.82
TY36	0.84	0.82	0.83	0.78	1.07	3.08	2.02	0.89
TY37	0.76	0.77	0.74	0.70	1.39	3.42	2.26	0.87
TY38	0.92	1.25	2.44	2.24	1.76	5.97	1.98	3.39
TY39	0.71	0.53	0.41	0.75	0.38	2.01	1.17	0.62
TY50	0.79	0.73	0.79	0.85	0.53	1.94	1.70	0.59
TY51	0.71	0.67	0.76	0.78	0.49	2.20	1.20	0.70
TY52	0.75	0.69	0.76	0.80	0.43	2.60	1.27	0.74
TY53	0.73	0.74	0.59	0.85	0.52	2.69	1.91	0.69
TY54	0.78	0.82	1.10	1.19	0.53	4.03	2.27	1.10

Table S5. The geoaccumulation index (I_{geo}).

No	Cr	Ni	Cu	Zn	As	Cd	Hg	Pb
TY01	−1.16	3.55	−0.16	1.64	−1.20	4.06	0.01	−0.52
TY02	−0.43	−0.29	0.80	0.70	0.80	2.48	1.67	1.22
TY03	−1.19	−1.20	0.19	−0.12	−0.74	2.64	0.90	0.74
TY04	−0.64	−1.17	−0.74	−0.62	−1.66	0.98	0.24	−0.72
TY05	−0.49	−0.42	−0.19	0.15	−1.15	1.45	0.58	−0.38
TY06	−0.30	−0.19	0.01	−0.22	−0.77	1.52	0.58	−0.43
TY07	−0.54	−0.70	−0.62	−0.56	−1.74	0.61	−0.15	−0.95
TY08	−0.16	−0.08	0.19	−0.17	−1.17	1.02	0.69	−0.45
TY09	−0.34	−0.48	−0.13	−0.27	−1.74	1.36	0.55	−0.17
TY10	−0.96	−1.16	−0.89	−0.93	−1.57	0.69	−0.01	−0.70
TY11	−0.18	−0.17	0.10	−0.25	−0.73	1.18	0.57	−0.63
TY12	−0.61	−0.73	−0.71	−0.53	−1.27	0.81	−0.17	−0.96
TY13	−0.75	−0.79	−0.45	−0.24	−1.44	1.19	0.10	−0.79
TY14	−0.64	−0.72	−0.35	−0.34	−1.34	1.77	0.04	−0.54
TY15	−0.50	−0.49	−0.10	−0.39	−1.07	1.52	0.44	−0.45
TY16	−0.21	−0.16	0.27	−0.06	−1.08	1.66	0.62	−0.30
TY17	−0.22	−0.63	−0.38	−0.31	−1.30	0.86	0.54	−1.16
TY18	−0.55	−0.93	−0.52	−0.73	−1.62	0.84	0.32	−0.97
TY19	−0.56	−0.82	−0.20	−0.51	−1.37	1.25	0.50	−0.83
TY20	−0.63	−0.78	0.04	−0.14	−1.26	1.33	0.61	−0.48
TY21	−0.85	−0.98	−0.71	−0.61	−1.26	1.05	1.04	−0.61

TY22	−0.74	−0.87	−0.69	−0.53	−1.68	1.02	−0.08	−0.72
TY23	−1.02	−1.23	−1.00	−1.11	−1.39	0.51	−0.62	−1.03
TY24	−1.01	−1.18	−1.11	−0.68	−1.56	1.18	0.01	−0.49
TY25	−0.73	−0.85	−0.37	−0.48	−1.38	1.10	0.37	−0.46
TY26	−0.22	−0.12	0.10	−0.20	−0.61	1.33	0.47	−0.56
TY27	−0.98	−1.37	−0.59	−0.56	−1.44	1.69	1.03	−0.06
TY28	−0.97	−1.27	−2.15	−1.27	−1.33	0.78	0.37	−0.64
TY29	−0.48	−0.72	−0.32	−0.47	−1.27	1.23	0.94	−0.83
TY30	−0.87	−0.89	−0.72	−0.78	−1.06	1.34	0.06	−0.62
TY31	−0.71	−0.47	0.11	−0.44	0.19	1.80	1.27	−0.11
TY32	−0.10	0.13	−0.01	−0.27	0.25	1.17	0.62	−0.76
TY33	−0.94	−0.83	−0.73	−0.80	−0.51	1.04	0.74	−0.81
TY34	−0.71	−0.81	−0.53	−0.55	−1.35	1.10	0.56	−0.61
TY35	−0.87	−1.00	−0.63	−0.62	−1.38	0.93	0.01	−0.77
TY36	−1.11	−1.15	−1.12	−1.21	−0.76	0.76	0.15	−1.03
TY37	−1.41	−1.39	−1.44	−1.51	−0.53	0.77	0.16	−1.22
TY38	−1.05	−0.61	0.36	0.23	−0.12	1.65	0.05	0.83
TY39	−0.54	−0.97	−1.34	−0.47	−1.45	0.96	0.17	−0.74
TY50	−0.74	−0.85	−0.75	−0.63	−1.32	0.56	0.36	−1.16
TY51	−0.71	−0.78	−0.60	−0.57	−1.25	0.93	0.05	−0.72
TY52	−0.66	−0.79	−0.64	−0.56	−1.46	1.13	0.09	−0.68
TY53	−0.99	−0.99	−1.30	−0.78	−1.48	0.89	0.38	−1.07
TY54	−0.70	−0.63	−0.19	−0.09	−1.24	1.68	0.84	−0.20
